# Influence of Weight Loss on Volumetric Change in Contralateral Breast During 2-Stage Breast Reconstruction

**Published:** 2018-09-27

**Authors:** Hirotaka Suga, Tomohiro Shiraishi, Akihiko Takushima

**Affiliations:** Department of Plastic Surgery, Kyorin University School of Medicine, Tokyo, Japan

**Keywords:** breast reconstruction, contralateral breast, volumetric change, body weight, weight loss

## Abstract

**Objective:** During 2-stage breast reconstruction in patients with unilateral breast cancer, we sometimes experience cases in which the contralateral breast volume changes greatly. However, few studies have examined volumetric changes in the contralateral breast during 2-stage breast reconstruction. **Methods:** Changes in contralateral breast volume between the first and second operations were examined in patients who underwent 2-stage unilateral breast reconstruction between February 2013 and August 2016 (123 patients aged 49.1 ± 8.6 years). Influences of age, postoperative treatment, and body weight on volumetric changes in the contralateral breast were statistically analyzed. **Results:** A positive correlation was observed between changes in body weight and contralateral breast volume (correlation coefficient = 0.218, *P* = .015). Weight loss was particularly important: all patients who lost more than 3 kg showed decreased contralateral breast volume (*P* = .010). Age and postoperative treatment had no significant effect on the change in contralateral breast volume. **Conclusion:** Change in body weight, and massive weight loss in particular, is an important factor for volumetric changes in the contralateral breast during 2-stage unilateral breast reconstruction.

Two-stage reconstruction with a tissue expander is currently the most common type of breast reconstruction. During 2-stage reconstruction in patients with unilateral breast cancer, we sometimes experience cases in which the contralateral breast volume changes greatly, requiring revision of reconstruction plans. Breast volume can be influenced by various factors such as aging,^1^ adjuvant therapy for breast cancer,^2^ and changes in body weight.^3^ However, few studies have examined volumetric changes in the contralateral breast during 2-stage breast reconstruction. In this study, we examined volumetric changes in the contralateral breast and analyzed the factors that influence these changes.

## PATIENTS AND METHODS

### Patients

All female patients who underwent 2-stage unilateral breast reconstruction between February 2013 and August 2016 at Kyorin University Hospital (123 patients aged 49.1 ± 8.6 years) were evaluated retrospectively. Breast volume was calculated on the basis of preoperative measurements of width, height, and projection, assuming that the breast was a quadrangular pyramid, as described previously.^4^ The anthropometric measurement in each patient was performed by the same surgeon before the first and second operations. Changes in the contralateral breast volume between the first and second operations were examined. Influences of age, postoperative treatment, and body weight on volumetric changes in the contralateral breast were statistically analyzed.

### Statistical Analysis

Data are expressed as means ± standard deviations. All analyses were performed using Statcel version 3. Comparisons between 2 groups were performed using the paired *t* test. Comparisons among multiple groups were performed using 1-way analysis of variance. Values of *P* < .05 were considered statistically significant.

## RESULTS

**Figure 1:**
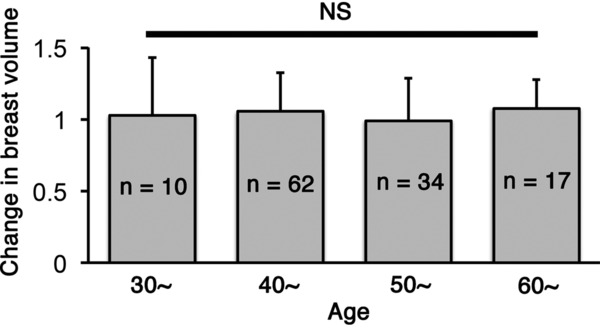
Changes in the contralateral breast volume in different age groups. There was no significant difference between the groups.

**Figure 2:**
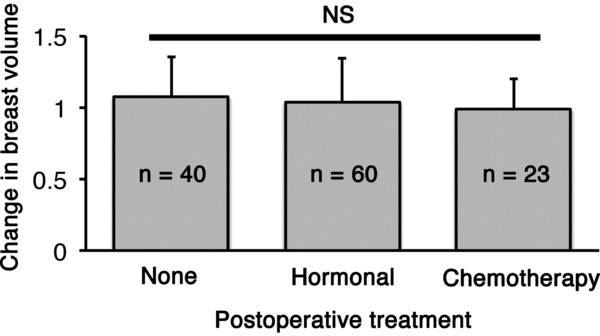
Changes in the contralateral breast volume in groups with different postoperative treatments. There was no significant difference between the groups.

**Figure 3:**
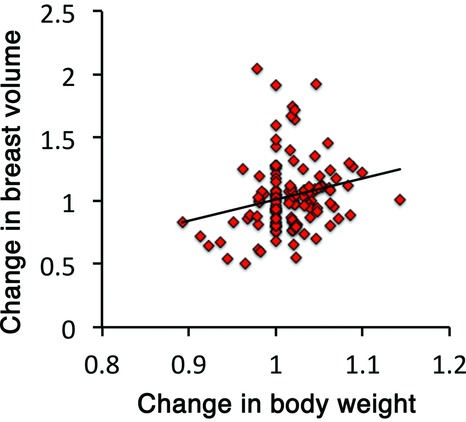
Correlation between the changes in body weight and contralateral breast volume (correlation coefficient = 0.218, *P* = .015).

**Figure 4:**
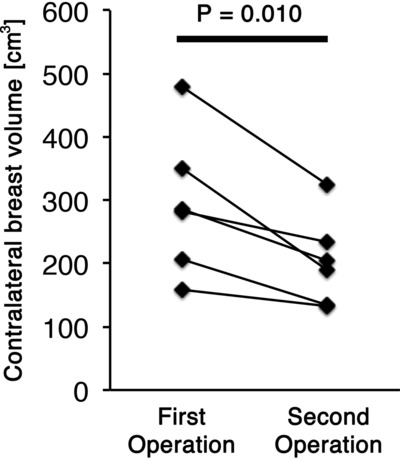
Volumetric changes in the contralateral breast in patients who lost more than 3 kg in body weight (*P* = .010).

**Figure 5:**
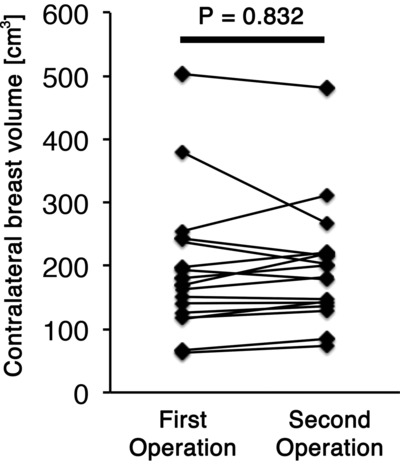
Volumetric changes in the contralateral breast in patients who gained more than 3 kg in body weight (*P* = .832).

**Figure 6:**
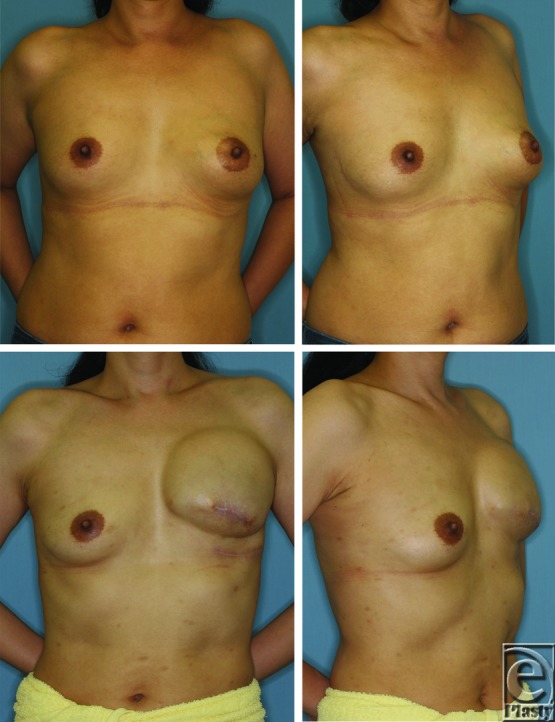
A 44-year-old woman with left breast cancer who underwent mastectomy and had no postoperative treatment. The patient had a body weight of 52 kg and contralateral breast volume of 207 cm^3^ before the first operation (upper panels). Before the second operation, the patient weighed 48 kg and the contralateral breast volume was 134 cm^3^ (lower panels).

**Figure 7:**
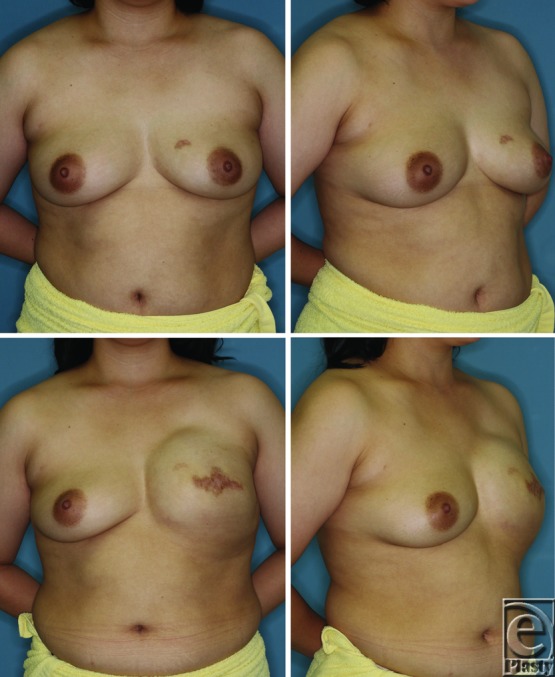
A 38-year-old woman with left breast cancer who underwent mastectomy and postoperative hormonal therapy. The patient had a body weight of 78 kg and contralateral breast volume of 480 cm^3^ before the first operation (upper panels). Before the second operation, the patient weighed 73 kg and the contralateral breast volume was 325 cm^3^ (lower panels).

The average interval between the first and second operations was 252 ± 68 days. The mean contralateral breast volume at the first and second operations was 180 ± 105 and 177 ± 101 cm^3^, respectively; the difference between them was not significant (*P* = .613). Age did not impact changes in the contralateral breast volume (*P* = .712; Fig 1). Postoperative treatments during the 2-stage breast reconstruction included hormonal therapy or chemotherapy; however, the type of treatment had no significant effect on changes in the contralateral breast volume (*P* = .505; Fig 2). A positive correlation was observed between changes in the body weight and the contralateral breast volume (correlation coefficient = 0.218, *P* = .015; Fig 3). The mean body weight at the first and second operations was 53.4 ± 7.7 and 54.1 ± 7.3 kg, respectively, and this increase was significant (*P* < .001). Interestingly, all patients who lost more than 3 kg in body weight showed decreased contralateral breast volume (*P* = .010; Fig 4), whereas patients who gained more than 3 kg in body weight did not show a significant change in the contralateral breast volume (*P* = 0.832; Fig 5). Representative cases with decreased contralateral breast volume are shown in Figures 6 and 7.

## DISCUSSION

In unilateral breast reconstruction, contralateral breast volume is important to achieve symmetric and aesthetically pleasing results.^5^ Contralateral breast volume affects the size of the implant in prosthetic reconstruction and the size of the flap in autologous reconstruction. Volumetric changes in the contralateral breast during 2-stage breast reconstruction may lead to a change in the scheduled size of the implant or flap. Our data showed that change in body weight, especially massive weight loss, is an important factor influencing volumetric changes in the contralateral breast. When we plan and select an implant or a flap for the second operation, we need to consider any change in body weight. Our data also imply that a change in body weight after the final operation could lead to a change in the contralateral breast volume and cause asymmetry of the reconstructed breast.

Breast volume has been measured by various methods, including anthropometric measurement,^6^^,^^7^ thermoplastic molding,^8^ computed tomography,^9^ and magnetic resonance imaging.^10^ Recent advances in technology have enabled 3-dimensional imaging of the breast and accurate measurement of breast volume.^11^^,^^12^ In our study, we calculated the breast volume using anatomical measurements of width, height, and projection, assuming that the breast was a quadrangular pyramid, as we described previously.^4^ Although this method is less accurate than imaging methods, we minimized the measurement error by ensuring that the same surgeon performed both preoperative measurements in each patient. Kayar et al^13^ reported an acceptable degree of accuracy in another anthropometric measurement with 4 factors (projection, medial radius, lateral radius, and inferior radius). Comparison of measurement methods for breast volume should be examined in the future study.

Another limitation of our study is that we did not analyze volumetric changes in the mammary gland. Ishii et al^2^ reported decreased contralateral breast volume due to adjuvant therapy, particularly in patients with high breast density. Although there were no significant differences between the groups with and without adjuvant therapy in our study, it is possible that a decrease in mammary gland volume occurred in the group with adjuvant therapy but was masked by increased subcutaneous tissue from weight gain. Assessment of the volume of the mammary gland and multivariate analyses for the change in the total breast volume are needed in the future.

It was interesting that patients who gained more than 3 kg in body weight did not, on average, show significant change in contralateral breast volume, whereas all the patients who lost more than 3 kg in body weight showed decreased contralateral breast volume. The data imply that subcutaneous tissue around the breast is more responsive to weight loss than that in other areas, although such specificity does not apply to weight gain. Vohra et al^3^ reported a significant reduction in breast volume after weight loss surgery. The responsiveness of subcutaneous tissue in the breast to change in body weight should be studied in detail in the future.
